# Oncogene Convergence in Extrachromosomal DNA Hubs

**DOI:** 10.1158/2159-8290.CD-22-0076

**Published:** 2022-04-10

**Authors:** Natasha E. Weiser, King L. Hung, Howard Y. Chang

**Affiliations:** 1 Center for Personal Dynamic Regulomes, Stanford University School of Medicine, Stanford, California.; 2 Department of Pathology, Stanford University School of Medicine, Stanford, California.; 3 Howard Hughes Medical Institute, Stanford University, Stanford, California.

## Abstract

Extrachromosomal DNA circles (ecDNA) are a common mechanism for oncogene amplification and are associated with worse clinical outcomes compared with other types of oncogene amplification. Several recent discoveries of ecDNA hubs—local congregations of ecDNAs in the nucleus—highlight unique features of ecDNA biology that may contribute to higher oncogene expression and rapid tumor evolution.

Oncogene amplifications are a common mechanism for tumorigenesis, granting selective advantages to cancerous cells by driving high-level expression of growth-promoting genes. Such amplifications can occur in various ways, such as in tandem repeats within chromosomes, termed homogeneously staining regions (HSR), or as extrachromosomal DNAs (ecDNA). ecDNAs are circular DNA structures ranging in size from approximately 100 kb to several megabases and are present in tens to hundreds of copies per cell (1, 2). One mechanism for ecDNA generation is chromothripsis (chromosome shattering; refs. 3, 4). It is possible that other mechanisms of DNA damage can contribute to ecDNA generation, although direct experimental evidence is lacking. While other types of DNA circles have been described in noncancerous cells, ecDNAs are distinguished from other DNA circles by their larger size, by their encoding of genes and regulatory elements, and by the fitness advantage that results from overexpression of ecDNA-encoded oncogenes. ecDNAs also lack centromeres and thus are subject to random segregation into daughter cells. Therefore within an ecDNA^+^ tumor, there may be a wide range of oncogene copy numbers that can promote tumor heterogeneity and facilitate the rapid adaptation of the tumor in the setting of targeted therapy (2, 5, 6). A recent analysis of data from The Cancer Genome Atlas and Pan-Cancer Analysis of Whole Genomes found enrichment of APOBEC3-mediated kataegis, or clustered mutagenesis events, on ecDNAs and in cancer genomes of ecDNA^+^ tumors (7). These findings suggest that within a single tumor, the variation in the ecDNA sequences as well ecDNA number might contribute to tumor heterogeneity.

ecDNAs include paired chromatin bodies termed double minutes (DM), first described in the 1960s (8, 9), although we now know that only a subset of ecDNAs are detectable as double minutes on chromosome spreads. With the advent of whole-genome sequencing and dedicated analytic pipelines, analyses of thousands of cancer samples have found that approximately 14% of primary tumor samples contain ecDNAs, including more than 50% of glioblastomas (1). Compared with cancers containing chromosomal oncogene amplifications, ecDNA-containing tumors are associated with worse patient outcomes and express a higher level of oncogene than would be expected on the basis of copy number alone (1, 2, 8). These observations raise the question of what intrinsic features of ecDNAs drive such high levels of gene expression. Recent studies have shown that the chromatin of ecDNAs is highly accessible to the transcriptional machinery, suggesting that epigenetic mechanisms may help to drive oncogene overexpression (10). However, our understanding of how the transcriptional dynamics of ecDNAs differ from chromosomal amplifications is incomplete.

Two recent studies published by our group and colleagues have demonstrated that in interphase cells, ecDNAs cluster together into micron-sized “hubs” that promote oncogene overexpression (11, 12). These studies identified ecDNA hubs in cells derived from multiple cancer types and harboring amplifications of *MYC, FGFR2*, and *EGFR* oncogenes, suggesting that ecDNA hubs represent a common mechanism by which ecDNAs can drive oncogene overexpression. Here, we highlight some of the key insights from these and other recent articles. For a comprehensive review of ecDNA biology, see ref. 13.

Utilizing a combination of DNA FISH analysis of fixed cells and two different methods for tracking ecDNAs in living cells, Hung, Yost, Xie, and colleagues (hereafter Hung and colleagues; ref. 11) and Yi and colleagues (12) demonstrated that during interphase, ecDNAs cluster into hubs that drive a higher level of gene expression than would be expected on the basis of copy number alone. These studies highlight the advantages of different approaches to tracking ecDNAs in live cells. Yi and colleagues (12) developed ecTag, a CRISPR–Cas9-based system where a single-guide RNA targeting sequence specific to ecDNA breakpoints is fused to 15 or 25 Pumilio binding sites and cotransfected with catalytically inactivated Cas9 (dCas9) and plasmids encoding a Clover–PUF fusion protein (12). With live-cell imaging, the ecTag system demonstrated the asymmetric segregation of ecDNAs into daughter cells after mitosis and the growth of ecDNA hubs during G_1_ phase of the cell cycle (12). However, ecTag was not able to visualize mitosis, perhaps because highly compacted mitotic chromatin is not accessible to dCas9 binding (12). Hung and colleagues (11) employed a different approach to visualize ecDNAs in live cancer cells. Insertion of a TetO array into the *MYC*-encoding ecDNAs and coexpression of TetR-GFP labeled the TetO-containing ecDNAs. With this method, Hung and colleagues (11) were able to visualize interphase ecDNA hubs, their dissolution into smaller structures during mitosis, and re-formation of ecDNA hubs after nuclear partitioning. The use of the TetO/TetR system has the advantage of allowing visualization of ecDNAs during mitosis (11). However, the TetO/TetR system requires genome editing to insert the TetO array and thus is laborious to use in multiple cell types and comes with the risk that a large insertion into the ecDNA may affect some aspects of ecDNA biology. The ecTag system takes advantage of the ecDNA breakpoint sequences, which are not present on chromosomal DNA, and does not require any genome editing; therefore, it may be easier to use across multiple cell types. Both methods for ecDNA tagging come with the caveat that not all ecDNAs within a given cell may be tagged and there is likely to be cell-to-cell variability in terms of the proportion of tagged ecDNAs, highlighting the importance of complementary approaches such as DNA FISH in fixed cells.

ecDNA hubs can also be observed by DNA FISH–targeting oncogenes in fixed cells. DNA FISH analysis identified hubs in multiple ecDNA^+^ human cancer cell lines, including COLO320-DM (*MYC*-amplified colorectal carcinoma), PC3 (*MYC*-amplified prostate cancer), HK359 (*EGFR*-amplified glioblastoma), and SNU16 (*FGFR2*- and *MYC*-amplified gastric cancer). By combined DNA and RNA FISH targeting the *MYC* locus and nascent *MYC* pre-mRNA, respectively, Hung and colleagues (11) found a significant correlation between the spatial clustering of ecDNAs and *MYC* pre-mRNA expression in COLO320-DM cells; in fact, ecDNA clustering was a better predictor of *MYC* pre-mRNA expression than ecDNA copy number. Yi and colleagues (12) independently identified ecDNA hubs in multiple *EGFR-*amplified glioblastoma neurosphere lines from both primary and recurrent tumors. They showed that RNA polymerase II (RNAPII) is more likely to colocalize with ecDNA hubs compared with actively transcribed, chromosomally encoded transcripts (12). Furthermore, larger ecDNA hubs are more colocalized with RNAPII compared with smaller hubs (12). These findings suggest that the clustering of ecDNAs into hubs is correlated with oncogene expression.

What is the mechanism by which the formation and size of hubs drive increased transcriptional activity? Hung and colleagues (11) turned to SNU16 gastric carcinoma cells, which contain two distinct ecDNA species: one encoding *MYC* and the other encoding *FGFR2*. Metaphase DNA FISH showed minimal overlap between *MYC* and *FGFR2*, confirming that they are in fact encoded on distinct ecDNA structures. During interphase, however, *MYC* and *FGFR2* DNA FISH signals colocalize in ecDNA hubs. Using high-throughput conformation capture with chromatin immunoprecipitation (HiChIP) targeting histone H3K27 acetylation (H3K27ac), a chromatin conformation method to enrich for chromatin interactions involving active enhancers and promoters (14), Hung and colleagues (11) found that *MYC* and *FGFR2* ecDNAs interact with each other in an intermolecular manner. Notably, both high-throughput conformation capture (Hi-C) and HiChIP analyses showed focal contacts with H3K27ac marks, suggesting that these intermolecular contacts may represent enhancer–gene interactions. These multiple sites of interaction between *MYC*- and *FGFR2*-encoding ecDNAs included five enhancers on the *FGFR2*-encoding ecDNAs that contacted the *MYC* promoter. To validate that these interactions promote oncogene transcription, Hung and colleagues (11) performed CRISPR interference (CRISPRi) to repress the enhancers on the *FGFR2*-encoding ecDNAs and observed a corresponding decrease in *MYC* expression. These findings show that interactions between ecDNAs within the hub facilitate enhancer–promoter interactions in *trans* to promote oncogene overexpression.

Intriguingly, intermolecular ecDNA interactions may not be limited solely to interactions among different ecDNA molecules. A recent report used a combination of chromatin confirmation and chromatin immunoprecipitation methods [Hi-C and RNAPII chromatin interaction analysis with paired-end tag (ChIA-PET)] to identify multiple interactions between enhancers on ecDNAs and chromosomal genes (15). In addition, they found that ecDNAs are enriched for super-enhancers (SE) and that chromosomal genes whose RNAPII-bound promoters made contact with ecDNAs were more highly expressed than genes with RNAPII-bound promoters that did not contact ecDNAs (16). These findings suggest that ecDNA-encoded enhancers and SEs interact with chromosomal promoters to promote the expression of chromosomally encoded genes. It is currently unclear whether ecDNA hubs or unclustered ecDNAs promote interactions between ecDNAs and chromosomal DNA or to what extent ecDNA hubs might contribute to broader transcriptional changes in cancer cells beyond those resulting directly from higher oncogene expression, but this is an important avenue for further investigation.

What protein factors facilitate the formation or maintenance of ecDNA hubs? The bromodomain-containing protein BRD4, an established regulator of canonical, chromosomally encoded *MYC* expression that is known to bind SEs that drive *MYC* expression, is a good candidate for a component of *MYC*-encoding ecDNA hubs. Using the live-cell imaging system described above, Hung and colleagues (11) discovered that epitope-tagged BRD4 colocalizes with *MYC*-encoding ecDNA hubs in COLO320-DM cells. Furthermore, chemical inhibition of bromodomain–chromatin interactions by treatment with the bromodomain inhibitor JQ1 disperses the ecDNA hubs. Live-cell imaging demonstrated the kinetics of ecDNA hub dispersal after bromodomain inhibition (∼20 minutes; ref. 11). Interestingly, at low concentrations of JQ1, *MYC* mRNA expression was significantly more sensitive to bromodomain inhibition in ecDNA^+^ cells (COLO320-DM) compared with an isogenic cell line containing chromosomal amplifications of *MYC* (COLO320-HSR). Insertion of a DNA element that strongly binds to BRD4 into a heterologous reporter gene plasmid is sufficient to recruit and promote transcription of the plasmid in ecDNA hubs in a bromodomain-dependent fashion. JQ1 treatment in SNU16 cells also caused decreased expression in both ecDNA-encoded oncogenes, *MYC* and *FGFR2*, consistent with a role for bromodomain proteins in maintaining ecDNA hubs to facilitate oncogene overexpression in multiple cell lines. In addition, live-cell imaging with labeled ecDNAs of COLO320-DM cells (as described above) showed that ecDNA hubs break into smaller clusters during mitosis and then recongregate in the daughter nuclei. However, when cells are treated with JQ1, ecDNA hubs do not re-form after mitosis, indicating that bromodomain proteins are required for both the formation and maintenance of ecDNA hubs in COLO320-DM cells (11).

It is currently unclear to what extent bromodomain proteins such as BRD4 might regulate ecDNA hubs in other cancer types with different amplified oncogenes. It may be that only some ecDNA-containing cancers—for example, those with *MYC*-encoding ecDNAs or a subset thereof—include bromodomain proteins in the hubs. We predict that although the identity of the hub-resident proteins may vary to some extent based on the oncogenes and/or enhancers in the hubs, the dependence of ecDNA hubs on particular chromatin-associated proteins is likely to be a conserved feature of ecDNA hubs. This presents the exciting possibility that the proteins required for hub maintenance may serve as potential drug targets. We saw direct evidence for this when comparing viability of COLO320-DM cells with the isogenic COLO320-HSR cells containing chromosomal *MYC* amplification after JQ1 treatment. We observed that COLO320-DM cells are significantly more sensitive to JQ1 treatment than COLO320-HSR cells, suggesting that tumor cells containing ecDNA hubs may be sensitized to targeted drug treatment compared with non–ecDNA-containing cells (11).

The findings discussed above have important implications for tumor heterogeneity. While ecDNA copy numbers are variable among cancer cells, gene products of amplified oncogenes are only modestly correlated with copy numbers (12). This observation suggests that there are other factors contributing to the variability of oncogene expression at the protein level (12). We posit that ecDNA hubs are a prime candidate for facilitating transcriptional variability. Chromatin conformation and single-cell joint RNA sequencing (RNA-seq) and assay of transposase-accessible chromatin by sequencing (ATAC-seq) demonstrate combinatorial enhancer–oncogene interactions on ecDNA and variable usage of enhancers among cells correlating with RNA expression (11). Live-cell imaging demonstrates that ecDNA hubs are dynamic structures with individual ecDNAs moving in and out of the hub as well as having unstable interactions with other nuclear structures such as PML and Cajal bodies (11, 12). These findings show that ecDNA hubs are dynamic. Given that RNAPII localization appears to correlate with larger hubs (12) and that hubs provide a mechanism for ecDNAs to “sample” and select for different enhancers (15, 17), the hubs may serve as a mechanism for fine-tuning the optimal level of oncogene expression. The recent finding that ecDNAs also interact extensively with some chromosomal loci to promote transcription of chromosomally encoded genes (15) suggests that the ecDNA hubs might also allow cancer cells to rapidly calibrate broader swaths of their transcriptomes beyond the genes encoded in the ecDNAs themselves. However, interactions between hubs and chromosomal loci have not yet been identified. The importance of ecDNAs for tumor heterogeneity and rapid evolution in response to cellular stress is highlighted by a recent preprint from our group and others (5).

To fully understand the roles of ecDNAs in cancer pathogenesis, we will need to explore many more aspects of ecDNA hub biology including but not limited to the following questions. First, the studies highlighted here found that ecDNA hubs are a shared feature of ecDNA^+^ tumors expressing *MYC*, *FGFR2*, and *EGFR*. We expect that many other ecDNA species also reside in hubs, although it is unclear whether hubs are truly a universal feature of ecDNA^+^ tumors. Second, in addition to bromodomain proteins, what other proteins reside in the hub and how might they facilitate hub formation or maintenance? To what extent are the protein components of the ecDNA hubs shared between cancer types and/or amplified oncogenes? Third, previous studies showed that ecDNAs cluster in response to DNA damage (18, 19)—are these clusters different from the hubs that we observe in interphase cells and could hubs play a role in DNA repair, for example, by recruiting repair proteins? Fourth, what is the role of RNA in an ecDNA hub? While ecDNA hubs are not sensitive to treatment with the transcriptional inhibitor alpha-amanitin, this finding does not address whether RNA may help to hold the hub together. Fifth, the impact of ecDNA segregation on intratumoral genetic heterogeneity, rapid evolution, selection, and treatment resistance remains to be delineated. These and additional questions will be active areas of investigation for future studies.
